# The Effects of Freshwater Clam (*Corbicula fluminea*) Extract on Serum Tumor Necrosis Factor-Alpha (TNF-α) in Prediabetic Patients in Taiwan

**DOI:** 10.3390/md20040261

**Published:** 2022-04-10

**Authors:** Tse-Hung Huang, Chiao-Hsu Ke, Chin-Chang Chen, Cheng-Hsun Chuang, Kuang-Wen Liao, Yi-Hsien Shiao, Chen-Si Lin

**Affiliations:** 1 Department of Traditional Chinese Medicine, Chang Gung Memorial Hospital, Keelung City 20401, Taiwan; huangtsehung@gmail.com; 2 School of Traditional Chinese Medicine, Chang Gung University, Taoyuan City 33302, Taiwan; 3 Graduate Institute of Integration of Western and Chinese Medicine Nursing, National Taipei University of Nursing and Health Sciences, Taipei City 23741, Taiwan; 4 Department & Graduate Institute of Chemical Engineering & Graduate Institute of Biochemical Engineering, Ming Chi University of Technology, New Taipei City 24301, Taiwan; 5 Graduate Institute of Health Industry Technology, Chang Gung University of Science and Technology, Taoyuan City 33303, Taiwan; 6 Department of Veterinary Medicine, School of Veterinary Medicine, National Taiwan University, No. 1 Sec. 4 Roosevelt Rd., Taipei City 10617, Taiwan; f08629002@ntu.edu.tw; 7 Department of Anatomy, School of Medicine, China Medical University, Taichung City 40402, Taiwan; geoge6211@gmail.com; 8 Institute of Molecular Medicine and Bioengineering, National Chiao Tung University, Hsinchu City 30068, Taiwan; charleschuang1993@gmail.com (C.-H.C.); liaonms@g2.nctu.edu.tw (K.-W.L.); 9 Institute of Molecular Medicine and Bioengineering, National Yang Ming Chiao Tung University, Hsinchu City 30068, Taiwan; 10 Drug Development and Value Creation Research Center, College of Dental Medicine, Kaohsiung Medical University, Kaohsiung 80708, Taiwan; 11 Center for Cancer Research, Kaohsiung Medical University, Kaohsiung 80708, Taiwan

**Keywords:** freshwater clam extract, *Corbicula fluminea*, tumor necrosis factor-alpha, prediabetes

## Abstract

Freshwater clam extract (FCE) is a functional food that regulates the immune system and has been demonstrated in numerous studies to display desirable anti–tumor necrosis factor-alpha (TNF-α) responses. In addition, excess TNF-α production is positively associated with type 2 diabetes. However, few longitudinal clinical studies evaluating the efficiency and toxicity of FCE are available. This article reports that patients with prediabetes who received FCE had a desirable outcome of a reduction in serum TNF-α for a long period. This was a double-blind, randomized, parallel clinical trial conducted using FCE intervention and placebo groups, and 36 patients with prediabetes were enrolled. Two grams of FCE or placebo was consumed daily for 180 consecutive days. The serum of the participants was collected at four time points (0M: before the intervention; 3M: after 3 months of intervention; 6M: after 6 months of intervention; 12M: 6 months after cessation of intervention at 6M). A serum TNF-α concentration higher than 4.05 pg/mL was defined as a cut-off value. FCE reduced serum TNF-α in all participants at 6M and 12M. Moreover, FCE significantly suppressed serum TNF-α concentrations at 6M and 12M and inhibited TNF-α release with time series in subjects with elevated TNF-α values. FCE intervention effectively reduced serum TNF-α and persistently sustained the effects for half a year in patients with prediabetes. Gas chromatography–mass spectrometry (GS-MS) analysis revealed that the major components of FCE were phytosterols and fatty acids, which exerted anti-inflammatory and anti-TNF-α abilities. Hence, FCE has the potential to be developed as a natural treatment for prediabetic patients in Taiwan.

## 1. Introduction

Prediabetes occurs before type 2 diabetes (T2D) and is a high-risk state for diabetes development [1]. It is projected that more than 470 million people will have prediabetes in 2030 [2]. Before developing full-blown diabetes, about 10% of patients will pass through the prediabetes phase [1], which is defined by the American Diabetes Association (ADA) as fasting glycemic levels that are higher than normal (100 mg/dL) but lower than the threshold of diabetes (125 mg/dL) [2,3]. Due to their elevated fasting glucose levels, these prediabetic patients have a high burden of cardiovascular risk, including hypertension [4]. Some treatments are recommended for prediabetes to prevent the progression of diabetes. First, lifestyle modification, such as dietary changes, has been suggested as the first-line approach in prediabetic patients [5]. Previous clinical trials showed that over a 3- to 6-year period, lifestyle interventions reduced the incidence of diabetes by 28–58% [6,7,8]. Furthermore, several clinical trials have been conducted to evaluate the benefits of various drug therapies for prediabetes/diabetes prevention, including troglitazone [9], liraglutide [10], metformin [8], and ramipril [11]. These trials have indicated that medical intervention reduces the diabetes risk by 25% to 79% compared with placebo. 

Prediabetic patients are highly associated with impairment of insulin secretion, insulin resistance (IR), and dysregulation of the incretin hormone [12,13], and TNF-α plays a vital role in this pathogenesis [14,15]. With the accumulation of TNF-α, glucose transporter type 4 (GLUT4), an insulin-regulated glucose transporter, will be downregulated [16], leading to increases in blood glucose levels [17]. In addition, the activation of TNF-α contributes to serine phosphorylation of insulin receptor substrate-1 (IRS-1) [16], resulting in the inability of IRS-1 to activate downstream phosphatidylinositol 3-kinase-dependent pathways [18]. GLUT4 and serine phosphorylation of IRS-1 are critical in the synthesis of insulin, and elevated TNF-α disturbs the function of these two essential enzymes. Likewise, TNF-α impairs the secretion of glucagon-like peptide-1 (GLP-1), an incretin hormone responsible for the amplification of insulin secretion [13]. Accumulation of TNF-α induces inflammation in pancreatic islets, leading to apoptosis of β-cells in pancreatic islets, and the impairment of pancreatic β-cells is the main causative factor in the suppression of insulin secretion [19,20]. Thus, persistent elevated TNF-α induces dysregulation of the incretin hormone, IR, and pancreatic β-cell dysfunction, which could accelerate the onset of diabetes [21]. 

Elevated TNF-α could result in the progression of diabetes [12,14,22,23]. The currently available anti-diabetes treatments act through various mechanisms against hyperglycemia, and many of these approaches also improve pancreatic β-cell function and IR, hence modifying the progression of diabetes [24]. Anti-TNF-α medicine or components may potentially prevent diabetes or prediabetes progression. Several nutritional supplements, such as FCE from *Corbicula fluminea*, a freshwater clam that is now known to also occur in brackish waters [25,26], are widely used as remedies against TNF-α. It is evidenced that FCE possesses numerous medical and biological advantages, including hepatic protection [27,28], anti-tumorigenesis properties [29,30], and anti-inflammatory activities [31,32]. FCE is a potential immune-regulatory agent because it can successfully suppress the release of a pro-inflammatory cytokine, TNF-α [28,33], and act as a functional food that can reduce cholesterol accumulation and attenuate inflammation [34]. Some nutritional supplements have been proposed instead of medical intervention to prevent diseases early [35,36]. Therefore, the main purpose of this study is to evaluate whether intervention with a nutritional extract, FCE, could reverse the progression of prediabetes and thereby slow the progression of prediabetic patients before the development of full-blown diabetes. Following ADA guidelines, this clinical trial recruited prediabetic volunteers, evaluated the effects of FCE on alleviating the serum TNF-α concentration, and monitored their physiology status for 12 months. 

## 2. Results

### 2.1. Demographics of Participants and Safety Evaluation of FCE in This Study

**Figure 1:**
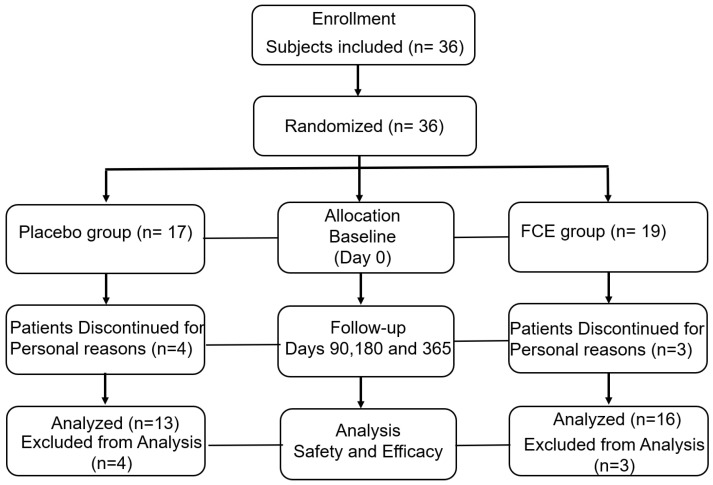
Flow diagram of the enrollment of patients with prediabetes. Subjects with prediabetes (*n* = 36) were initially enrolled and randomly allocated into two groups. Because of personal consideration, four and three participants withdrew from this clinical trial in the control and FCE groups, respectively. During the 12-month intervention, paired serums of patients with prediabetes were collected at the beginning and on days 90, 180, and 365 to evaluate the efficiency and safety of FCE.

In this study, 36 Taiwanese patients were initially enrolled and randomly allocated into placebo (*n* = 17) and FCE groups (*n* = 19). A detailed flow chart of the study is shown in Figure 1. In the placebo group, the 17 patients included 12 women (70.59%) and 5 men (29.41%) with a median age of 54.47 years. Their average BMI (kg/m^2^), height (cm), and weight (kg) were 24.8 ± 3.84, 157.81 ± 8.51, and 61.9 ± 11.19, respectively. The FCE group comprised 19 patients, including 12 females (63.2%) and 7 males (36.8%), with a median age of 52.95 years and an average height of 159.47 cm ± 6.88 cm. Though there were no significant differences in weight and BMI, the participants in the FCE group were heavier (average: 66.6 kg ± 13.54) and had higher BMI (26.01 ± 3.85) than those in the other group. Collectively, the gender (*p* = 0.1052), age (*p* = 0.6405), height (*p* = 0.5231), body weight (*p* = 0.2731), and BMI (*p* = 0.3545) of all volunteers initially had no significant differences between the two groups. The demographics are shown in Table 1. To evaluate the safety and efficacy of FCE in participants, a thorough physical examination and creatinine (CRE), blood urea nitrogen (BUN), alanine aminotransferase (ALT), aspartate aminotransferase (AST), uric acid (UA), and albumin (ALB) tests were performed to determine general health status at 0, 3, 6, and 12 months. There were no significant differences in these blood examinations, and no severe side effects were observed (Figure S1 and Table 2). ALB was the majority of total serum protein and represented the nutritional condition of the participants, as hypoalbuminemia is related to malnutrition or other illness. ALT, AST, and UA were the indicators of hepatic functions, and renal functions were evaluated by CRE and BUN. Interestingly, serum TNF-α concentrations were significantly decreased in subjects consuming FCE as compared with the placebo group (*p* < 0.001; Table 2).

### 2.2. FCE Significantly Reduced Serum TNF-α Concentration of All Participants during and after Nutritional Intervention

**Figure 2:**
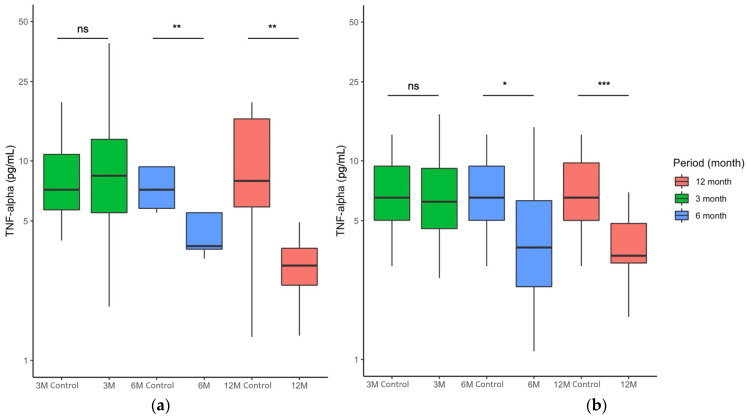
Comparison of serum TNF-α concentrations among various time points after the consistent 6-month intervention. Paired tests of serum TNF-α in all subjects receiving (**a**) placebo or (**b**) FCE after 3, 6, and 12 months are shown in a boxplot. Control values at 3M, 6M, and 12M indicate the paired serum TNF-α in the same prediabetic patients at the initial time point (0M). Data are presented as median ± interquartile range. ns, no significant difference; *, *p* < 0.05; **, *p* < 0.01; ***, *p* < 0.001.

As TNF-α concentrations significantly improved in prediabetic patients consuming FCE, we further evaluated the alteration of serum TNF-α concentrations in all participants. Serum TNF-α values in subjects receiving placebo were 9.04 ± 7.63 after 3 months of intervention, while the values were 8.56 ± 12.85 initially, suggesting no difference in the first 3 months (*p* > 0.05; Figure 2a, columns in green). There was a temporary decrease in serum TNF-α concentration at 6 months with a median value of 4.43 ± 11.67 compared to the median value of 8.56 ± 13.65 in the beginning (*p* < 0.01; Figure 2a, columns in blue). Interestingly, participants taking the placebo ultimately exhibited a decreased median value of 2.99 ± 2.35 in serum TNF-α concentration, as the median TNF-α concentration was 8.56 ± 12.61 (*p* < 0.01; Figure 2a, columns in red). These findings indicated that the placebo might slightly impact the TNF-α concentration, as we speculated that the component of the placebo (microcrystalline cellulose) possibly harbors an anti-inflammatory ability. In the FCE group, the mean values of TNF-α concentration did not change obviously in the first 3 months; the mean values were 5.69 ± 4.63 and 6.72 ± 4.20 with or without FCE consumption (*p* > 0.05; Figure 2b, columns in green). After 6 months of intervention, the TNF-α concentration obviously declined from 6.72 ± 4.20 to 2.37 ± 3.43 (*p* < 0.05; Figure 2b, columns in blue). Notably, although the participants received no FCE for an additional 6 months, the serum TNF-α concentration continued to decrease from 6.72 ± 4.73 to 1.64 ± 4.08 (*p* < 0.001; Figure 2b, columns in red). These results indicated that FCE not only effectively alleviated the serum TNF-α concentration but also steadily improved serum TNF-α in all participants for half a year after the withdrawal of the nutritional supplement.

### 2.3. FCE Significantly Improved Patients with Elevated Serum TNF-α Concentrations during and after Nutritional Intervention

**Figure 3:**
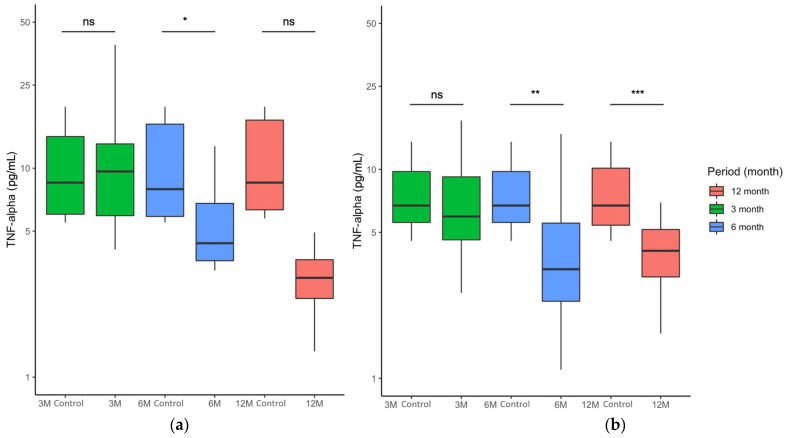
Comparison of serum TNF-α concentrations at various time points after the consistent 6-month intervention. Figures show paired analysis of serum TNF-α in patients with serum TNF-α concentrations above 4.05 pg/mL who consumed (**a**) placebo or (**b**) FCE after 3, 6, and 12 months in a boxplot. Control values at 3M, 6M, and 12M indicate the paired serum TNF-α in the same prediabetic patients at the initial time point (0M). Data are presented as median ± interquartile range. ns, no significant difference; *, *p* < 0.05; **, *p* < 0.01; ***, *p* < 0.001.

FCE successfully improved the progression of serum TNF-α in all individuals (Figure 2). To investigate whether FCE alleviated the serum TNF-α release in patients with a higher serum TNF-α concentration, participants whose serum TNF-α concentrations were above 4.05 pg/mL were selected for further assessment. At first, the median values of the placebo and FCE groups were 9.29 ± 13.16 and 6.72 ± 4.20, respectively. After 3 months of intervention, whether the prediabetic patients with higher TNF-α values received placebo (10.51 ± 7.93; Figure 3a, columns in green) or FCE (5.69 ± 4.63; Figure 3b, columns in green) had no impact on their serum TNF-α concentrations (*p* > 0.05). Though the placebo temporarily affected the amount of TNF-α in the sixth month (5.12 ± 14.02 pg/mL) compared to the control (9.41 ± 13.38; *p* < 0.05; Figure 3a, columns in blue), it predictably failed to decrease the TNF-α concentration in prediabetic patients throughout the trial (3.11 ± 2.43 pg/mL) compared to the initial time point (9.17 ± 12.95; *p* > 0.05; Figure 3a, columns in blue). On the other hand, the TNF-α values were 2.37 ± 3.43 after 6 months of FCE consumption, lower than those of the control group (6.72 ± 4.20). Furthermore, after the withdrawal of FCE intake, the TNF-α values continuously decreased to 1.64 ± 4.08 pg/mL (compared to control values of 6.72 ± 4.73). These results indicated that FCE significantly inhibited the levels of TNF-α after consistent consumption for 6 months (*p* < 0.01; Figure 3b, columns in blue) and even stably reduced the release of this pro-inflammatory cytokine into circulation for at least 6 months after cessation of consumption (*p* < 0.001; Figure 3b, columns in red). To sum up, these findings indicated that FCE effectively stabilized the serum TNF-α concentration during the intervention. After 6 months of continuous consumption, FCE constantly improved physical health and potentially relieved the progression of diabetes.

### 2.4. Sequential Correlation of Serum TNF-α Concentration and Time Exhibited in the Patients with Prediabetes Receiving FCE

**Figure 4:**
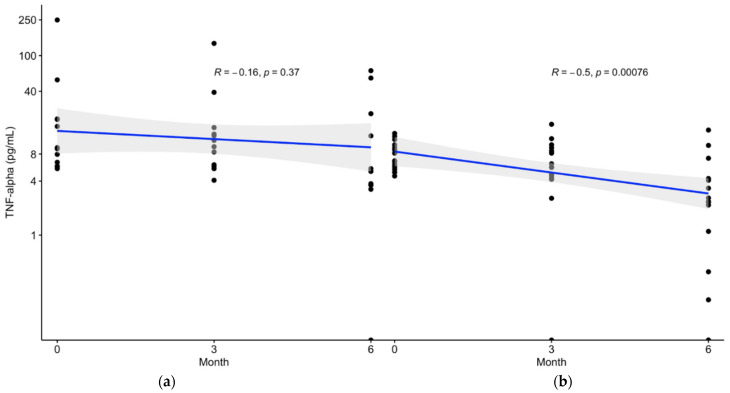
Correlation of timepoints and serum TNF-α concentration after the 6-month intervention. (**a**) Placebo did not successfully reduce serum TNF-α concentrations of participants with subhealth status at 6 months (*p* = 0.37); (**b**) FCE not only inhibited serum TNF-α concentration (*p* = 0.00076) but also significantly alleviated TNF-a progression (*R* = −0.5) in patients with subhealth status (*R* = −0.16). Data were considered to be significantly different if the *p* value was less than 0.05. TNF-α, tumor necrosis factor-alpha; FCE, freshwater clam extract.

FCE inhibited the release of TNF-α into circulation for 6 and 12 months in all individuals and patients with TNF-α concentrations initially above 4.05 pg/mL. To determine the correlation between serum TNF-α concentration and time series in the continuous intervention, patients with elevated TNF-α concentrations were selected for sequential analysis between these two indicators. Placebo had little impact on reducing serum TNF-α concentration (*p* = 0.3700; Figure 4, left panel), but FCE significantly inhibited the TNF-α concentration (*p* = 0.0001; Figure 4, right panel) of patients with prediabetes during the intervention. Furthermore, FCE more effectively reduced TNF-α progression (*R* = −0.5; Figure 4, right panel) than did the placebo (*R* = −0.16; Figure 4, left panel). The sequential correlation of TNF-α concentrations during the continuous-time points (0M, 3M, and 6M) indicated that FCE could effectively suppress pro-inflammatory cytokines in prediabetic patients with time series.

### 2.5. A Detailed Analysis of FCE Reveals That the Major Bioactive Compounds Derived from FCE Were Highly Associated with Anti-TNF-α Effects

**Figure 5:**
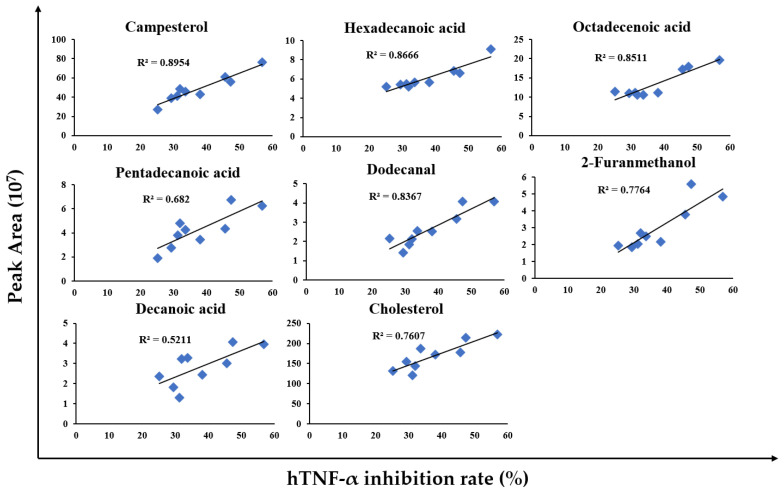
Phytosterols and fatty acids derived from *Corbicula fluminea* effectively regulate the inflammatory responses. Identified bioactive compounds can significantly inhibit TNF-α secretion. THP-1 cells were treated with LPS (1 µg/mL) and FCE and the supernatants were collected. The selected samples were submitted to GC-MS for component analysis, and the abundant target compounds (peak area, *Y*-axis) were positively correlated (*R* square > 0.5) with the TNF-α inhibition rate (*X*-axis).

In our previous study, we used gas chromatography–mass spectrometry (GC-MS) to identify the components of FCE, which can be divided into phytosterols (PSs) and fatty acids [23]. Herein, to determine the potential mechanisms that significantly reduced the TNF-α in patients, we subsequently investigate the major components of FCE and their correlation with anti-TNF-α effects using GC-MS. Figure 5 shows the bioactive compounds of FCE that can significantly reduce TNF-α. A human leukemia monocytic (THP-1) cell line was treated with lipopolysaccharides (LPSs) to elicit the secretion of TNF-α. After FCE treatment, the inflammatory responses were determined by enzyme-linked immunosorbent assay (ELISA), which showed that FCE could reduce the secretion of TNF-α by 25–57% compared to the LPS stimulation alone (Figure 5, *X*-axis, human tumor necrosis factor-alpha (hTNF-α) inhibition rate). The FCE was subsequently submitted to compound analysis by GC-MS. The abundance of effective anti-inflammatory agents (Figure 5, *Y*-axis, peak area), namely campesterol, hexadecanoic acid, octadecenoic acid, pentadecanoic acid, dodecanal, 2-furanmethanol, decanoic acid, and cholesterol, was positively correlated with the anti-inflammatory activities (*R*^2^ > 0.5). These results indicated that FCE exhibits desirable anti-TNF-α effects in a dose-dependent manner, which may potentially improve the health of prediabetic patients. 

## 3. Discussion

This study assessed the correlation of serum TNF-α in prediabetic patients following FCE consumption, and it also evaluated the safety, long-term efficacy, and potential mechanisms of this nutritional supplement. Prediabetic individuals have relatively insufficient immune systems, and FCE has been widely used as a functional food against these symptoms [27,28]. FCE successfully suppressed the release of TNF-α in our previous in vitro and in vivo studies [23]. Herein, we have further evaluated the effect of FCE in suppressing the TNF-α concentration in clinical trials, and the results indicate that FCE significantly inhibits TNF-α and provides long-term protection in patients with prediabetes. 

In the prediabetic patients who consumed FCE in this study, serum TNF-α was effectively reduced during the intervention and persistently decreased over the subsequent 6 months, as shown in Figure 2 and Figure 3. Despite the lack of a significant decrease, the stable changes in glucose (Figure S2a) and glycated hemoglobin (Figure S2b) concentrations until the end of this trial suggest that FCE can stabilize glucose-related indicators and limit the progression toward full-blown diabetes. We also evaluated the safety and long-term effects of FCE. As shown in Table 2 and Figure S1, long-term consumption of FCE does not lead to severe side effects such as hepatic failure (evaluated by ALT and AST), renal toxicity (evaluated by CRE and BUN), dysfunction of nutrition (evaluated by ALB), or gout (evaluated by UA), as evidenced by the insignificant changes in these biochemistry analyses. The long-term intake of FCE not only has no toxicity but also effectively suppresses TNF-α with time series in prediabetic patients with serum TNF-α concentrations above 4.05 pg/mL. A significantly negative correlation between TNF-α concentration and FCE intake is shown in Figure 4, highlighting the fact that long-term consumption of FCE by patients with prediabetes leads to more obvious inhibitory effects of TNF-α. These results indicate that FCE safely reduces serum TNF-α in circulation and potentially lowers the progression of diabetes in a time-dependent manner for at least 6 months.

Our results strongly indicate that FCE derived from *Corbicula fluminea* can suppress the release of TNF-α in patients with prediabetes. These effective anti-TNF-α effects should result from the major components, such as campesterol and octadecenoic acid (oleic acid), which were identified by GC-MS. The major components of FCE (Figure 5) can be divided into PSs and fatty acids [23]. PSs present anti-inflammatory properties and reduce lipid levels, thereby improving several chronic inflammatory diseases [37,38]. The PSs and campesterol exerted the inhibitory effects of infiltration of 12-O-tetradecanoylphorbol-13-acetate (TPA)-induced inflammatory murine models [39]. The fatty acids in FCE, such as octadecenoic acid, could effectively regulate inflammation by blocking the nuclear factor-kappa B (NF-κB) pathway [40] and inhibiting the expression of inducible nitric oxide synthase (iNOS) and cyclooxygenase-2 (COX-2) [41]. Therefore, FCE can modulate inflammation and even harbors antioxidant, immunomodulatory, antibacterial, and antifungal effects [42,43]. In addition to the effect of reducing TNF-α in this study, the bioactivities of the clam extract can alleviate decreased hepatic function, increased blood pressure, and accumulated cholesterol [35,36]. FCE can suppress the release of fatty acid synthase (FAS), a central enzyme in lipogenesis, thereby leading to a hepatoprotective function and a decrease in hepatic triacylglycerol [44]. Furthermore, herbal extracts have a function similar to that of an angiotensin-converting enzyme (ACE) inhibitor, a substance that contributes to lower blood pressure [45]. FCE can reverse liver injury by reducing cholesterol accumulation and improving dysregulated cholesterol synthesis [34]. Most importantly, the anti-inflammatory effects of FCE can attenuate the progression of various inflammatory diseases, such as chronic hepatitis [31,33]. In the current study, FCE is shown to stabilize TNF-α concentration and suppress the release of one pro-inflammatory cytokine, interleukin-1 beta (IL-1β), which induces COX-2 expression and prostaglandin E2 (PGE2) formation [46]. The extract possesses anti-tumor properties as well, inducing apoptosis in human gastric cancer cells [30] and promoting cell death in leukemia cells [29]. FCE can induce intracellular glutathione depletion, reactive oxygen species production, and coordinative modulation of the Bcl-2 family in cancer cells, leading to apoptosis of these neoplastic cells [29]. Many bioactivities of herbal extracts, such as hepatoprotection [27,28], antihypertension [45], anti-tumorigenesis [29,30], and hypocholesterolemia [34], have been reported. Collectively, FCE has been shown not only to suppress TNF-α concentration but also to act as a functional food in a variety of studies, indicating that FCE is a potential immunoregulator that can serve as a functional supplement in prediabetic patients.

Though the native habitat of *C. fluminea* is originally in the lakes, rivers, and streams in Asia and Africa, it is now found in both freshwater and saltwater throughout North America, including all five Gulf states and northern Mexico, and much of Europe [47]. It was also reported that almost 90% of *C. fluminea* can survive in a saltwater solution of 70 g/L with 60 min immersion (seawater: approximately 35 g/L), whereas 80% and 90% of clams will die when immersed in 2% Virkon for 5 min and 10% bleach for 60 min, respectively [48]. Furthermore, one previous study also indicated that *C. fluminea* is an invasive species and is more resistant to saltwater compared to other invasive species, including perch (*Perca fluviatilis*), koi carp (*Cyprinus carpio carpio*), and tench (*Tinca tinca*) [49]. By investigating the health benefits of *C. fluminea*, this study also provides a potential solution to this invasive alien species via encouraging consumers to increase the ingestion of *C. fluminea* or clam-based supplements. 

To the authors’ knowledge, this was the first large-scale clinical study in which a natural anti-TNF-α agent was given to patients with prediabetes in Taiwan. In contrast to previous studies, the current study involved the recruitment of volunteers in the clinic and monitoring of their body conditions and changes in TNF-α concentrations for a long duration. A significant reduction of the serum TNF-α concentration was observed in participants following a 6-month FCE intervention and an additional 6 months of health status monitoring. In the comparison with placebo, we observed no significant influence of FCE on physiological functions. In conclusion, this clinical study illustrated that FCE effectively inhibits the TNF-α concentration in circulation and followed up on the physical statuses of all participants for a long period. Furthermore, FCE appears to be applicable as a natural treatment for other inflammatory diseases and to have the potential to be developed as an immunomodulator in clinical medicine in the future.

## 4. Materials and Methods

### 4.1. Subjects and Study Design

This study was conducted at the Chang Gung Memorial Hospital in Taipei, Taiwan, from May 2017 to June 2020. Thirty-six patients with prediabetes were enrolled. All were aged between 25 and 75 years and diagnosed with prediabetes according to the following criteria: glucose AC of 100–125 mg/dL, hemoglobin A1c (HbA1c) of 5.7–6.4%, and total cholesterol above 160 mg/dL or low-density lipoprotein cholesterol (LDL-C) above 100 mg/dL. Patients were excluded if they met the following criteria: (1) pregnancy or lactation; (2) poor medication compliance; (3) abnormal liver function (ALT and AST values above 2 fold-change of the upper reference value); (4) abnormal renal function (creatinine value above 1.5 mg/dL); (5) abnormal gastrointestinal function (such as gastrostomy, enterostomy, and diarrhea); (6) severe comorbidity in the last 6 months (such as brain stroke, myocardial infarction, major trauma or surgery); (7) prescribed medicine that may influence blood sugar, blood pressure, and blood lipids (such as hormones, corticosteroids, H2 blockers, diuretics, and statins); (8) diabetes; (9) other severe diseases (e.g., malignant tumors and Alzheimer’s disease); (10) current inflammatory diseases, infectious diseases, and severe immune deficiency (such as tuberculosis, acquired immune deficiency syndrome, active pneumonia, systemic lupus erythematosus, and rheumatoid arthritis); (11) other endocrine diseases that had impacts on blood sugar (such as hyperthyroidism, acromegaly, Cushing’s syndrome, and pheochromocytoma).

This was a double-blind, randomized, parallel, placebo-controlled clinical trial conducted using intervention and placebo groups. Participants completed written informed consent before the trial and were allocated by permuted-block randomization into two groups: FCE and placebo groups. A detailed flow chart of the study is shown in Figure 1. The intervention allocation was blinded for both investigators and participants. The study was approved by the Ethics Committee of Chang Gung Memorial Hospital and was registered on the American Registry of Clinical Trials website (http://ClinicalTrials.gov, accessed on 1 March 2017, identifier: NCT04429737).

### 4.2. Preparation of Freshwater Clam Extract (FCE)

The FCE was provided by Zhao Hong Biotechnology Co., Ltd. (Taipei, Taiwan). The shells of the freshwater clams were opened by boiling them in water, and the edible portions were removed immediately and extracted with hot water. The edible portions were harvested and freeze-dried. Then, they were ground and examined to be used as FCE. The FCE yield from raw material was approximately 2% (*w*/*w*). The approximate composition of 100 g of FCE is protein, carbohydrate, moisture, crude fat, and ash, which are 62.8%, 6.9%, 4.0%, 20.9%, and 5.4%, respectively. The crude extracted powder from FCE was stored at −20 °C until use in the experiment. The FCE powder was analyzed by SGS Taiwan. 

### 4.3. FCE Intervention

FCE was used in the form of capsules, and based on a human trial carried out in prediabetic patients, a dose of 2 g was chosen. Patients were advised to take two capsules before their meals day and night for 180 days. FCE and placebo capsules were obtained from Zhao Hong Biotechnology Co., Ltd. (Taipei, Taiwan). One experimental capsule contained 500 mg FCE, and each placebo capsule contained 500 mg microcrystalline cellulose, which was the same as the formulation component of the FCE capsule. The shape and color of the FCE and placebo capsules were identical. Compliance with the study was evaluated by telephone contact every week to ask the patients about their capsule consumption. Returned capsules were counted at the middle and end of the study. 

### 4.4. Anthropometric Parameters, Biochemistry Assays, and TNF-α Measurement

Weight and height were measured on an auto-anthropometer (super-view, HW-3080, Taiwan) with subjects wearing light clothing, and body mass index (BMI) was calculated by dividing the weight (kg) by the height squared (m^2^). Blood samples were taken from each subject by peripheral venipuncture after 12 h fasting at the baseline and at 3, 6, and 12 months. Blood samples were separated by centrifugation at 3000 rpm for 10 min at 4 °C to obtain the serum and frozen at −80 °C for further processing. Fasting serum glucose (Glucose AC), ALT, AST, uric acid (UA), creatinine (CRE), blood urea nitrogen (BUN), and albumin (ALB) in the serum were determined with the Cobas C501 analyzer (Roche Ltd., Mannheim, Germany), using commercial kits (Roche Ltd., Germany). Glycated hemoglobin (HbA1c) was measured by high-performance liquid C\chromatography (HPLC) (Trinity Biotech, Iran). For cytokine measurement, a human TNF-α DuoSet ELISA kit (R&D Systems, Minneapolis, MN , USA) was used following the manufacturer’s instructions. 

### 4.5. Gas Chromatography–Mass Spectrometry Analysis (GC-MS)

The GC-MS analysis of FCE was carried out at the National Yang Ming Chiao Tung University (Hsinchu, Taiwan) using a Hewlett-Packard 5890 II GC (J&W DB-5MS column, 30 m × 0.25 mm i.d.; film thickness 0.25 µm) coupled to a TRIO-2000 Micromass spectrometer (Micromass, Beverly, MA, USA). The carrier gas was pure helium (99.999%) at a constant flow rate of 1 mL/min. The injection temperature was set at 300 °C. The oven temperature was programmed from 50 to 310 °C at 10 °C/min, held isothermal for 10 min, and finally raised to 310 °C at 10 °C/min. Diluted samples (1/100, *v*/*v*, in methanol) of 0.5 µL were injected in the split mode with a split ratio of 10:1. The relative percentages of the chemical constituents in crude extract were expressed as a percentage by peak area normalization. 

### 4.6. Cell Culture, LPS Stimulation, and Cytokine Measurement

THP-1 cells were obtained from the Food Industry Research and Development Institute (BCRC, Hsinchu, Taiwan) and cultured in RPMI 1640 medium with GlutaMAX (Gibco, Carlsbad, CA, USA), supplemented with 10% fetal bovine serum (Gibco, Carlsbad, CA, USA), 100 U/mL of penicillin and 100 μg/mL streptomycin (Simply, Taipei, Taiwan), 20 mM HEPES solution (Sigma, St. Louis, MO, USA), and 20 nM 2-mercaptoethanol (Gibco, Carlsbad, CA, USA), and incubated in 5% CO_2_ at 37 °C. THP-1 cells (2 × 10^5^) were seeded in 24-well plates with 1 µg/mL LPS (Sigma, USA) and incubated for 24 h. Following FCE treatment (100 µg/mL), cells were centrifuged (5 min, room temperature, and 3000 rpm) and the supernatants were harvested and assayed using a human TNFα ELISA kit (R&D System, Minneapolis, MN, USA). 

### 4.7. Statistical Analysis

The statistical analysis was performed using R software 3.6.0 (26 April 2019) with several statistical packages. The ggplot2 package was used to create all of the plots. The missing data were excluded before each analysis. For descriptive statistics, tables were created with the xtable package. Wilcoxon signed-rank test in ggpubr package was used to compare quantitative and qualitative variables for paired non-parametric analysis. Spearman’s method was used for evaluating the correlation coefficient between concentration (pg/mL) and time (month) with the ggscatter function. All results with *p* values of less than 0.05 were considered statistically significant. 

## 5. Conclusions

Prediabetic patients who received the FCE intervention had significant declines in serum TNF-α, and the effect was persistently sustained for half a year. This was a large-scale clinical trial that recruited volunteers consuming nutritional supplements and monitored the physiological statuses of all subjects in Taiwan. Taken together, the findings of this study indicate that FCE has the potential to be developed as an anti-TNF-α functional food.

## Figures and Tables

**Table 1:** Baseline demographics of participants in this clinical study. There were no significant differences in sex, age, height, weight, or BMI between the placebo and experimental groups (*p* > 0.05). Data were considered to be significantly different when the *p* value was less than 0.05.

Variable	Placebo Group		Freshwater Clam Extract Group		*p* Value
	(*n* = 17)		(*n* = 19)		
	*n*	%	*n*	%	
Sex					0.1052
Female	12	70.59	12	63.16	
Male	5	29.41	7	36.84	
Age, years (SD)	54.47 (10.61)		52.95 (8.78)		0.6405
25–45	2	11.76	5	26.32	
45–65	13	76.47	12	63.16	
over 65	2	11.76	2	10.53	
Height, cm (SD)	157.81 (8.51)		159.47 (6.88)		0.5231
Weight, kg (SD)	61.90 (11.19)		66.60 (13.54)		0.2731
BMI, kg/m^2^ (SD)	24.80 (3.84)		26.01 (3.85)		0.3545
<18.5	0	0	0	0	
18.5–23.9	7	41.18	7	36.84	
24–26.9	5	29.41	5	26.32	
27–29.9	3	17.65	4	21.05	
30–34.9	2	11.76	3	15.79	
≥35	0	0	0	0	

**Table 2:** Characteristics of participants with placebo or FCE powder consumption. TNF-α values are shown as median (interquartile), and other data are presented as mean (SD).

	Placebo Group					Freshwater Clam Extract Powder Group				
	0 Months	3 Months	6 Months	12 Months	*p* Value	0 Months	3 Months	6 Months	12 Months	*p* Value
	*n* = 17	*n* = 17	*n* = 16	*n* = 13		*n* = 19	*n* = 19	*n* = 19	*n* = 16	
TNF-α, pg/mL	8.56 (12.85)	9.04 (7.63)	4.43 (11.67)	2.99 (2.35)	0.784	6.31 (4.28)	5.96 (4.71)	2.48 (2.91)	3.06 (3.71)	<0.001
ALT, U/L	24.1 (10.4)	23.2 (10.4)	23.2 (10.2)	28.8 (18.0)	0.587	34.5 (32.7)	33.6 (28.7)	32.8 (23.4)	32.1 (41.4)	0.996
AST, U/L	22.1 (4.79)	20.0 (3.91)	20.8 (4.97)	22.9 (7.62)	0.443	23.7 (8.44)	24.2 (9.10)	22.7 (7.99)	21.1 (9.49)	0.744
UA, mg/dL	5.18 (1.54)	5.23 (1.56)	5.40 (1.75)	5.48 (1.88)	0.955	5.88 (1.70)	6.00 (1.69)	5.69 (1.60)	5.47 (1.40)	0.790
CRE, mg/dL	0.74 (0.15)	0.73 (0.15)	0.76 (0.18)	0.72 (0.16)	0.942	0.81 (0.23)	0.84 (0.24)	0.82 (0.21)	0.80 (0.22)	0.957
BUN, mg/dL	15.1 (3.81)	14.5 (4.32)	14.7 (3.24)	14.9 (3.45)	0.972	14.2 (3.66)	14.5 (2.93)	15.0 (3.07)	14.5 (2.95)	0.893
ALB, g/dL	4.47 (0.30)	4.41 (0.27)	4.54 (0.28)	4.44 (0.41)	0.669	4.56 (0.22)	4.60 (0.24)	4.55 (0.24)	4.48 (0.32)	0.526

## Data Availability

The data presented in this study are available on request from the corresponding author.

## Supplementary Materials

The following supporting information can be downloaded at: https://www.mdpi.com/article/10.3390/md20040261/s1, Figure S1: Dynamic biochemical data of participants for evaluation of safety and toxicity of FCE powder, Figure S2: Dynamic glucose and HbA1c concentrations of participants for evaluation of FCE powder.
