# Supplementary material for: The Effects of Freshwater Clam (Corbicula fluminea) Extract on Serum Tumor Necrosis Factor-Alpha (TNF-α) in Prediabetic Patients in Taiwan
Source: Mar Drugs. 2022 Apr 10;20(4):261. doi: 10.3390/md20040261 (PMC9027770; doi:10.3390/md20040261)
Supplement: Supplementary file 1 [file marinedrugs-20-00261-s001.zip › marinedrugs-1636272-supplementary.pdf]

Supplementary Information

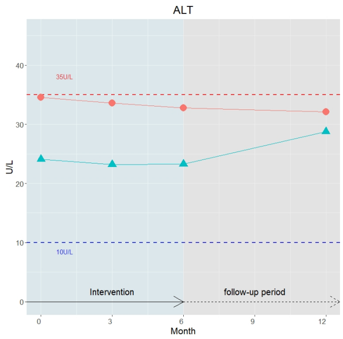

(**a**)

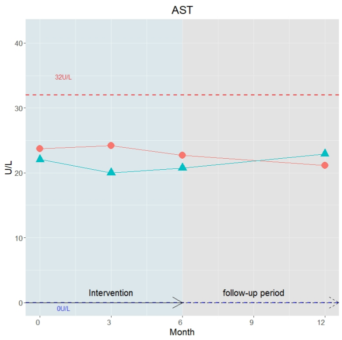

(**b**)

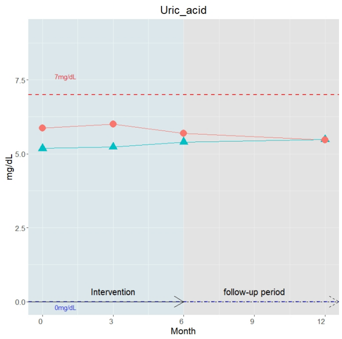

(**c**)

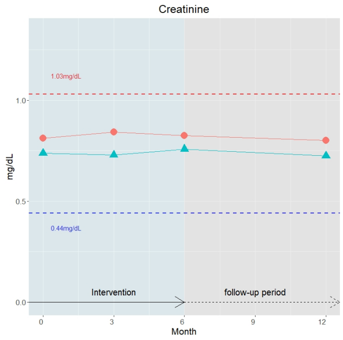

(**d**)

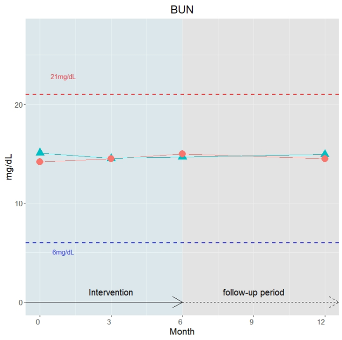

(**e**)

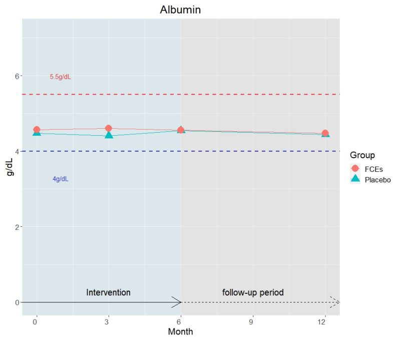

(**f**)

Figure S1. Dynamic biochemical data of participants for evaluation of safety and toxicity of FCE powder. Laboratory examinations were performed at 0, 3, 6 and 12 months after start of consumption of FCEs or placebo. (**a**) ALT (U/L), (**b**) AST (U/L), and (**c**) UA (mg/dL) for evaluation of hepatic function did not differ within the 6-month intervention or follow-up period. Renal functions were assessed by (**d**) CRE (mg/dL) and (**e**) BUN (mg/dL), and the alteration of these two indicators was also not significant. (**f**) ALB (g/dL) did not change between these two groups either. The horizontal dashed line shows the reference value. FCEs, freshwater clam extracts; ALT, alanine aminotransferase; AST, aspartate aminotransferase; UA, uric acid; CRE, creatinine; BUN, blood urea nitrogen; ALB, albumin.

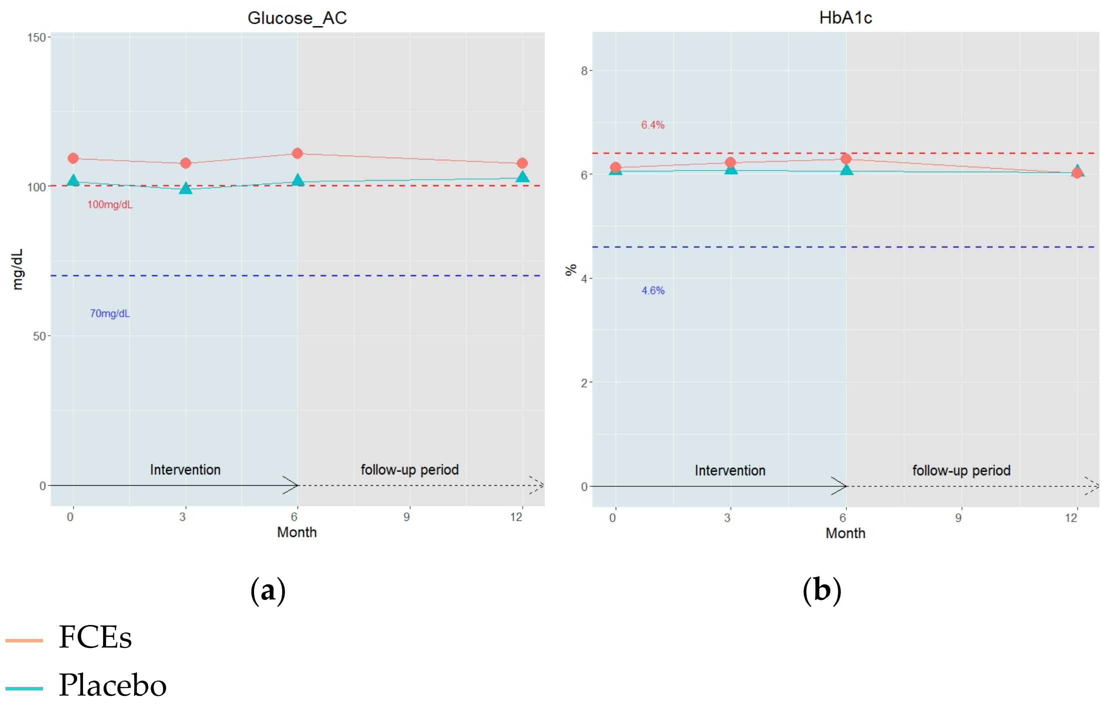

Figure S2. Dynamic glucose and HbA1c concentrations of participants for evaluation of FCE powder. Laboratory examinations were performed at 0, 3, 6 and 12 months after start of consumption of FCEs or placebo. (**a**) Glucose (mg/dL) and (**b**) HbA1c (%) did not differ within the 6-month intervention or follow-up period. The horizontal dashed line shows the reference value. FCEs, freshwater clam extracts; HbA1c, glycated hemoglobin.
